# LUMiC^®^ Endoprosthetic Reconstruction After Periacetabular Tumor Resection: Short-term Results

**DOI:** 10.1007/s11999-016-4805-4

**Published:** 2016-03-28

**Authors:** Michaël P. A. Bus, Andrzej Szafranski, Simen Sellevold, Tomasz Goryn, Paul C. Jutte, Jos A. M. Bramer, M. Fiocco, Arne Streitbürger, Daniel Kotrych, Michiel A. J. van de Sande, P. D. Sander Dijkstra

**Affiliations:** 1 Department of Orthopaedic Surgery, Leiden University Medical Center, Albinusdreef 2, 2300 RC Leiden, The Netherlands; 2 Institute of Mother & Child, Warsaw, Poland; 3 Oslo University Hospital, Oslo, Norway; 4 Maria Sklodowska-Curie Memorial Cancer Center and Institute of Oncology, Warsaw, Poland; 5 University Medical Center Groningen, Groningen, The Netherlands; 6 Academic Medical Center, Amsterdam, The Netherlands; 7 Department of Medical Statistics and Bioinformatics, Leiden University Medical Center, Leiden, The Netherlands; 8 Mathematical Institute, Leiden University, Leiden, The Netherlands; 9 Universitätsklinikum Münster, Münster, Germany; 10 Pomeranian Medical University, Szczecin, Poland

## Abstract

**Background:**

Reconstruction of periacetabular defects after pelvic tumor resection ranks among the most challenging procedures in orthopaedic oncology, and reconstructive techniques are generally associated with dissatisfying mechanical and nonmechanical complication rates. In an attempt to reduce the risk of dislocation, aseptic loosening, and infection, we introduced the LUMiC^®^ prosthesis (implantcast, Buxtehude, Germany) in 2008. The LUMiC^®^ prosthesis is a modular device, built of a separate stem (hydroxyapatite-coated uncemented or cemented) and acetabular cup. The stem and cup are available in different sizes (the latter of which is also available with silver coating for infection prevention) and are equipped with sawteeth at the junction to allow for rotational adjustment of cup position after implantation of the stem. Whether this implant indeed is durable at short-term followup has not been evaluated.

**Questions/purposes:**

(1) What proportion of patients experience mechanical complications and what are the associated risk factors of periacetabular reconstruction with the LUMiC^®^ after pelvic tumor resection? (2) What proportion of patients experience nonmechanical complications and what are the associated risk factors of periacetabular reconstruction with the LUMiC^®^ after pelvic tumor resection? (3) What is the cumulative incidence of implant failure at 2 and 5 years and what are the mechanisms of reconstruction failure? (4) What is the functional outcome as assessed by Musculoskeletal Tumor Society (MSTS) score at final followup?

**Methods:**

We performed a retrospective chart review of every patient in whom a LUMiC^®^ prosthesis was used to reconstruct a periacetabular defect after internal hemipelvectomy for a pelvic tumor from July 2008 to June 2014 in eight centers of orthopaedic oncology with a minimum followup of 24 months. Forty-seven patients (26 men [55%]) with a mean age of 50 years (range, 12–78 years) were included. At review, 32 patients (68%) were alive. The reverse Kaplan-Meier method was used to calculate median followup, which was equal to 3.9 years (95% confidence interval [CI], 3.4–4.3). During the period under study, our general indications for using this implant were reconstruction of periacetabular defects after pelvic tumor resections in which the medial ilium adjacent to the sacroiliac joint was preserved; alternative treatments included hip transposition and saddle or custom-made prostheses in some of the contributing centers; these were generally used when the medial ilium was involved in the tumorous process or if the LUMiC^®^ was not yet available in the specific country at that time. Conventional chondrosarcoma was the predominant diagnosis (n = 22 [47%]); five patients (11%) had osseous metastases of a distant carcinoma and three (6%) had multiple myeloma. Uncemented fixation (n = 43 [91%]) was preferred. Dual-mobility cups (n = 24 [51%]) were mainly used in case of a higher presumed risk of dislocation in the early period of our study; later, dual-mobility cups became the standard for the majority of the reconstructions. Silver-coated acetabular cups were used in 29 reconstructions (62%); because only the largest cup size was available with silver coating, its use depended on the cup size that was chosen. We used a competing risk model to estimate the cumulative incidence of implant failure.

**Results:**

Six patients (13%) had a single dislocation; four (9%) had recurrent dislocations. The risk of dislocation was lower in reconstructions with a dual-mobility cup (one of 24 [4%]) than in those without (nine of 23 [39%]) (hazard ratio, 0.11; 95% CI, 0.01–0.89; p = 0.038). Three patients (6%; one with a preceding structural allograft reconstruction, one with poor initial fixation as a result of an intraoperative fracture, and one with a cemented stem) had loosening and underwent revision. Infections occurred in 13 reconstructions (28%). Median duration of surgery was 6.5 hours (range, 4.0–13.6 hours) for patients with an infection and 5.3 hours (range, 2.8–9.9 hours) for those without (p = 0.060); blood loss was 2.3 L (range, 0.8–8.2 L) for patients with an infection and 1.5 L (range, 0.4–3.8 L) for those without (p = 0.039). The cumulative incidences of implant failure at 2 and 5 years were 2.1% (95% CI, 0–6.3) and 17.3% (95% CI, 0.7–33.9) for mechanical reasons and 6.4% (95% CI, 0–13.4) and 9.2% (95% CI, 0.5–17.9) for infection, respectively. Reasons for reconstruction failure were instability (n = 1 [2%]), loosening (n = 3 [6%]), and infection (n = 4 [9%]). Mean MSTS functional outcome score at followup was 70% (range, 33%–93%).

**Conclusions:**

At short-term followup, the LUMiC^®^ prosthesis demonstrated a low frequency of mechanical complications and failure when used to reconstruct the acetabulum in patients who underwent major pelvic tumor resections, and we believe this is a useful reconstruction for periacetabular resections for tumor or failed prior reconstructions. Still, infection and dislocation are relatively common after these complex reconstructions. Dual-mobility articulation in our experience is associated with a lower risk of dislocation. Future, larger studies will need to further control for factors such as dual-mobility articulation and silver coating. We will continue to follow our patients over the longer term to ascertain the role of this implant in this setting.

**Level of Evidence:**

Level IV, therapeutic study.

## Introduction

Surgical treatment of pelvic bone tumors continues to pose a challenge to the orthopaedic oncology community. Traditionally, pelvic tumors were resected by means of hindquarter amputation, a procedure associated with detrimental cosmetic, physical, and psychological outcomes [19]. At present, the majority of patients can be treated with limb-salvaging internal hemipelvectomies [19, 32]. Complications nevertheless remain frequent, especially for resections comprising the periacetabulum (Enneking Type 2 or Type 2–3) [8, 12, 14], and for large tumors, which are common in this location because pelvic tumors regularly attain large sizes before diagnosis. Procedures in this location also can be complicated by inadequate margins and, because the procedures are long, infection [3, 15].

Apart from tumor resection, obtaining a well-functioning reconstruction is challenging. As a result of the frequently massive extent of bone and soft tissue resection, the reconstructions are typically exposed to high biomechanical stresses. Reconstructive techniques remain a topic of debate; various biological, mechanical, and combined techniques have been advocated [4, 7, 10, 31]. Disadvantages of biological reconstruction using allografts, include the high risk of infection, nonunion, and graft resorption [5]. Many authors therefore consider endoprosthetic replacement a better solution to achieve satisfactory and durable functional and cosmetic results [15, 28, 33]. Several new implants have been introduced during recent decades, including custom-made, saddle, and “inverted ice cream cone” or “pedestal cup” prostheses [7, 11, 15, 20, 24, 28]. Most of these have been associated with a disappointing frequency of mechanical complications and failures, especially in the long term, including (recurrent) dislocations (3%–24%), aseptic loosening (3%–15%), cranial migration, heterotopic ossification, and periprosthetic or prosthetic fractures [5, 7, 11, 24, 25, 28]. However, adequately comparing different techniques is difficult because most published results are derived from single-center case series with limited patient numbers.

**Fig. 1:**
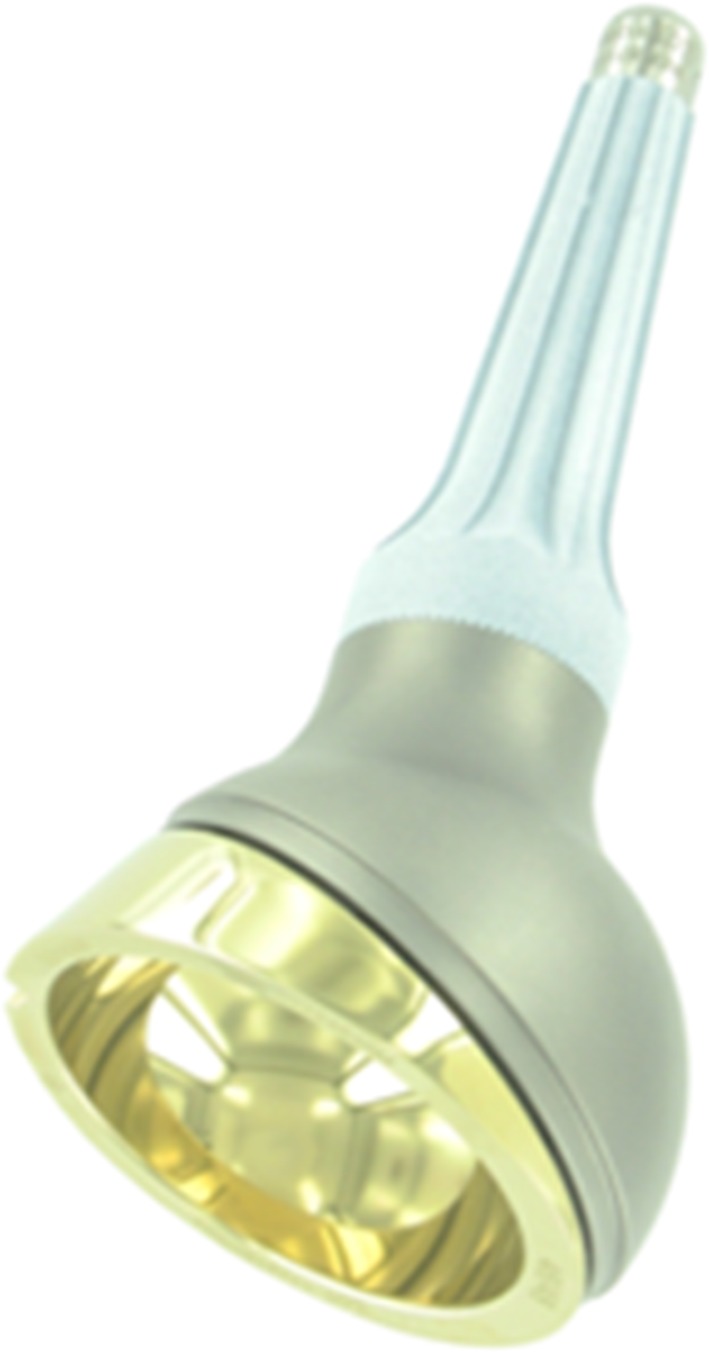
The LUMiC^®^ prosthesis consists of a separate cup and stem, both available in different sizes and with different coatings. Reproduced with permission from implantcast.

In the leading center of the current study, a pedestal cup prosthesis (Zimmer, Freiburg, Germany) was used for periacetabular reconstruction between 2003 and 2008 [7]. We encountered frequent complications, but considered the basic concept behind the implant suitable because it allows for relatively easy, quick, and durable fixation. Moreover, it allows for pelvic reconstruction even if only the medial ilium remains. We theorized that modification of the implant would aid to reduce complication rates and incorporated these ideas in the design of the LUMiC^®^ (implantcast, Buxtehude, Germany). The LUMiC^®^ prosthesis is a modular device, built of a separate stem (hydroxyapatite [HA]-coated uncemented or cemented) and acetabular cup (Fig. 1). The stem and cup are available in different sizes (the latter of which is also available with silver coating for infection prevention) and are equipped with sawteeth at the junction to allow for rotational adjustment of cup position after implantation of the stem. We hypothesized that aforementioned features would lead to a lower risk of aseptic loosening, dislocation, and infection and better restoration of lower limb function. The current study was initiated to evaluate the short-term clinical results of this implant.

Specifically, we asked: (1) What proportion of patients experience mechanical complications and what are the associated risk factors of periacetabular reconstruction with the LUMiC^®^ after pelvic tumor resection? (2) What proportion of patients experience nonmechanical complications and what are the associated risk factors of periacetabular reconstruction with the LUMiC^®^ after pelvic tumor resection? (3) What is the cumulative incidence of implant failure at 2 and 5 years and what are the mechanisms of reconstruction failure? (4) What is the functional outcome as assessed by Musculoskeletal Tumor Society (MSTS) score at final followup?

## Materials and Methods

**Fig. 2:**
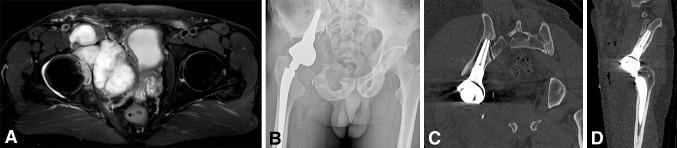
**A**–**D** (**A**) Case discussion of a 44-year-old male patient. T2-weighted MR image in the transverse plane shows a bulky mass, originating in the right acetabulum and infiltrating the hip joint. CT-guided biopsy showed a Grade 2 chondrosarcoma. (**B**) Conventional radiograph displaying the situation after Type 2–3 internal hemipelvectomy and subsequent reconstruction. Reconstruction was performed with an uncemented LUMiC^®^ stem (75 mm long, 10-mm core diameter), a 54-mm outer diameter HA-coated cup, and an uncemented Mallory-Head total hip prosthesis (Biomet, Warsaw, IN, USA) with a 28-mm femoral head. (**C**) CT scan displaying the position of the LUMiC^®^ stem in the coronal plane with its tip close to the sacroiliac joint. (**D**) CT scan displaying the position of the LUMiC^®^ stem in the sagittal plane.

Longitudinally maintained institutional registries were reviewed in eight centers of orthopaedic oncology to identify patients who underwent reconstruction with the LUMiC^®^ after periacetabular hemipelvectomy for a pelvic tumor. We reviewed every patient in whom this implant was used for this indication from July 2008 to June 2014 with a minimum followup of 24 months. The LUMiC^®^ was the preferred technique for reconstruction of pelvic defects after en bloc resection of a periacetabular tumor in all centers during the period under study. Alternative treatments included hip transposition and saddle or custom-made prostheses in some centers; these were generally used when the medial ilium was involved in the tumorous process or if the LUMiC^®^ was not yet available in the specific country at that time. Our general indications for using the LUMiC^®^ were reconstruction of periacetabular defects after pelvic tumor resections in which the medial ilium (adjacent to the sacroiliac joint, part 1A according to a modified version of Enneking’s classification [7]) was preserved, allowing the stem to be properly inserted (the conical stem is designed to seat between the anterior and posterior cortices of the medial part of the iliac wing, adjacent to the sacroiliac joint [Fig. 2]).

Forty-seven patients (26 males [55%]) with a mean age of 50 years (range, 12–78 years) were included (Table 1). At review, 32 patients (68%) were alive and 15 (32%) had died (nine of disease). Two patients with a metastatic tumor were referred to their local hospital and died within 2 years. The contributing center checked with their local hospital; no revisions or reoperations were undertaken before they died. One patient was lost to followup before 2 years and was excluded. The reverse Kaplan-Meier method was used to calculate median followup, which was equal to 3.9 years (95% confidence interval [CI], 3.4–4.3). Fifteen patients were treated in Center 1; other centers had seven, six, five, four, four, four, and two patients, respectively. The indication for pelvic resection was a primary bone tumor in 38 patients (81%; predominantly conventional chondrosarcoma; n = 22 [47%]), osseous metastases of distant carcinoma in five (11%), multiple myeloma with acetabular destruction in three (6%), and acetabular metastases of a previously resected femoral osteosarcoma in one (2%). Whether patients with metastatic disease were candidates for a pelvic resection and prosthetic reconstruction depended on the extent of acetabular destruction, patient prognosis (based on tumor type, Karnofsky performance score, and the presence of visceral or brain metastases), and morbidity. The technical feasibility of a limb-salvaging resection and subsequent reconstruction was assessed in multidisciplinary teams preoperatively.

**Table 1:** Study data

Variable	Number	Percent
Sex		
Male	26	55
Female	21	45
Indications for primary reconstructions		
Chondrosarcoma Grade 2 or 3	13	28
Metastatic carcinoma	5	11
Osteosarcoma	5	11
Ewing’s sarcoma	4	9
Chondrosarcoma Grade 1	4	9
Multiple myeloma	3	6
Pleomorphic undifferentiated sarcoma	1	2
Sarcoma not otherwise specified	1	2
Phosphaturic mesenchymal tumor	1	2
Indications for revision procedures (original diagnosis in parentheses)		
Pedestal cup reconstruction (two Grade 2 chondrosarcomas, one clear cell chondrosarcoma)	3	6
THA (Grade 2 chondrosarcoma)	1	2
Internal hemipelvectomy (P2) reconstructed with massive pelvic allograft and THA (Grade 2 chondrosarcoma)	1	2
Total femoral replacement (osteosarcoma)	1	2
THA and Müller cage (chondroblastoma)	1	2
Partial resection of iliac wing (P1) (dedifferentiated chondrosarcoma)	1	2
Partial resection of periacetabulum (P2) reconstructed with femoral head interposition (Grade 2 chondrosarcoma)	1	2
Resection type (Enneking classification)		
Type 2–3	26	55
Type 2	21	45
Neoadjuvant and adjuvant therapies		
Neoadjuvant chemotherapy	17	36
Adjuvant chemotherapy	12	26
Neoadjuvant radiotherapy	7	15
Adjuvant radiotherapy	10	21
Surgical details		
Extraarticular resections	20	43
Computer-assisted resections	12	26
MUTARS^®^ attachment tube used	16	34
Complications		
Dislocations, all reconstructions	10	21
Dislocations in primary dual-mobility cups (n = 24)	1	4
Structural complications	3	6
Infection	14	30
Local recurrence	5	11
Failure		
Any reason	8	17
Status at final followup		
No evidence of disease	29	62
Alive with disease	3	6
Died of disease	9	19
Died of other cause	6	13

The resections were Type 2 in 21 patients (45%) and Type 2–3 in 26 (55%). Twenty patients (43%) had an extraarticular resection. Nine patients (19%) had surgery before the LUMiC^®^ reconstruction, including three Pedestal cup™ reconstructions (6%; all had failed as a result of infection) and two allograft reconstructions (4%; one failed as a result of graft resorption, one as a result of local recurrence) (Table 1).

The LUMiC^®^ was designed for periacetabular reconstruction after tumor resection or extensive revision hip arthroplasty. It is a modular device built of a separate stem and cup, which are both equipped with sawteeth at the junction to allow for rotational adjustment of cup position after implantation of the stem (Fig. 1). The stem is hexagonally shaped and carries two additional wings to secure rotational stability. Stems are available for uncemented (TiAl_6_V_4_, HA-coated) and cemented (CoCrMo) fixation in three different lengths (65, 75, and 85 mm) and two different core diameters (8 and 10 mm, the latter only uncemented). Uncemented fixation was preferred in all centers unless bone quality was deemed insufficient or adequate press-fit fixation could not be obtained. The cups come in three different sizes (50, 54, and 60 mm outer diameter), uncoated, HA-coated, or silver-coated (only the 60-mm version). The highly crosslinked polyethylene inserts (implacross^®^; implantcast) are available in a neutral version and with 4-mm offset. The ACCIS^®^ liner (Accis BV, Baarn, The Netherlands) was first used in 2010 and offers the possibility of dual-mobility articulation when combined with the Polaric femoral head (implantcast).

Tumor resections were planned on an array of conventional imaging, CT, and MRI. Patients were positioned in the lateral decubitus position, allowing them to be rotated to nearly prone or supine positions. Before surgery, patients received intravenous cephalosporin antibiotics; these were usually continued for 1 to 5 days. Eighteen patients (38%) received tranexamic acid. The surgical approach and technique depended on the surgeon and tumor location. After resection, a cannulated probe was introduced in the remaining ilium; fluoroscopy or computer navigation was used to make sure the iliac cortices were not perforated. Use of computer navigation (n = 15 [32%]) depended on center preferences. A Kirschner wire was inserted through the probe, after which the ilium was reamed and a trial shaft was inserted. Next, the femoral component was implanted according to appropriate procedures. The cup was connected to the trial stem and a trial reduction was performed. After assessment of reconstruction length and soft tissue tension, the definitive stem was impacted (or cemented) and the cup was connected; a second trial reduction was then performed. Attachment (Trevira) tubes (implantcast) were used to reattach soft tissues and to stimulate neocapsule formation in 16 (34%) reconstructions [17]. Twenty-four patients (51%) had a dual-mobility cup; these were mainly used in case of a higher presumed risk of dislocation in the early period of our study. Later, dual-mobility cups became the standard for the majority of the reconstructions. Silver-coated acetabular cups were used in 29 reconstructions (62%); its use depended on the cup size that was chosen, because only the largest cup size was available with silver coating (Table 2). The iliac stem was cemented in four (9%; two multiple myelomas, one metastatic carcinoma, one chondrosarcoma). Twenty-three patients (49%) had standard hip prostheses and 23 (49%) had proximal femoral replacements; one patient (2%) had a previously implanted total femoral arthroplasty.

**Table 2:** Details of prosthetic components

Variable	Number	Percent
*LUMiC* ^®^ *stem size (uncemented, unless otherwise stated)*		
65 mm, 8 mm Ø	5	11
65 mm, 10 mm Ø	9	19
75 mm, 8 mm Ø	1	2
75 mm, 8 mm Ø, cemented	2	4
75 mm, 10 mm Ø	11	23
85 mm, 8 mm Ø	6	13
85 mm, 10 mm Ø	13	28
*LUMiC* ^®^ *cup size (outer Ø)*		
50 mm	6	13
54 mm	12	26
60 mm	29	62
*Femoral component*		
Cemented	12	26
Standard total hip prosthesis	24	51
Proximal femoral replacement	22	47
Total femoral replacement	1	2
*Femoral head size and articulation*		
28 mm, dual-mobility	16	34
32 mm	2	4
32 mm, dual-mobility	1	2
36 mm	21	45
36 mm, dual-mobility	7	15

Adequate margins were obtained in 39 of the 41 procedures (95%) intended to achieve clear margins; tumor spill occurred in two (5%; one clear cell chondrosarcoma, one phosphaturic mesenchymal tumor). Six patients (13%) had intentional intralesional surgery (five metastatic carcinomas, one chondroblastoma).

Usually, full weightbearing mobilization was started on the third postoperative day under supervision of a physical therapist. We used a rehabilitation protocol that is identical to that used in patients with revision hip arthroplasty. Starting from Day 3, partial weightbearing with two crutches is allowed until 6 weeks postoperatively. Thereafter, patients start to mobilize with one crutch. We believe it is important to mobilize patients as soon as possible to lessen the likelihood of major complications such as thrombosis. In the first days of mobilization, patients exercise for 1 to 2 hours and stay in bed during the remaining hours. Median postoperative hospital stay was 16 days (range, 4 days to 2.8 months). Routine followup included physical examination and radiographic and functional evaluation at 1 and 6 weeks; at 3 (conventional radiographs), 6 (conventional radiograph and CT), 12, and 24 months (conventional radiographs, CT and MRI); and yearly thereafter (conventional radiographs, MRI).

Medical records were evaluated to obtain characteristics of the patient, tumor, resection, and reconstruction. In consultation with the leading author (MPAB), one physician involved in the care of the patients in each center collected the data. Complications were classified according to Henderson et al. [22]. Aseptic loosening and periprosthetic and prosthetic fractures were diagnosed on imaging or intraoperatively. Aseptic loosening was defined as migration of the implant on conventional radiographs or CT or halo formation on CT in the absence of infection. Infection was defined as any deep (periprosthetic) infectious process diagnosed by physical examination, imaging, laboratory tests (C-reactive protein, erythrocyte sedimentation rate, leukocyte count), and microbiologic cultures. The occurrence of local recurrences was determined on imaging (usually MRI) and on histopathology in case surgery was performed. Failure was defined as removal or revision of (part of) the implant for any reason.

### Statistical Analysis

A competing risks model was used to estimate the cumulative incidence of implant failure for mechanical failure and infection with patient mortality as a competing event [26, 29]. A Cox regression model was used to study the effect of prognostic factors on survival. Categorical variables were compared between groups with chi-square tests and numerical variables with Mann-Whitney U tests. Outcomes are expressed in hazard ratios (HRs), 95% CIs, and p values. Functional outcome was assessed with the 1993 version of the MSTS questionnaires [13] at last followup; questionnaires were available for 24 patients (51%). Statistical analysis was performed using SPSS 21.0 (IBM Corp, Armonk, NY, USA) with the level of significance at p < 0.05.

## Results

A total of 30% (14 of 47) of our patients experienced one or more mechanical complications. A single dislocation (Henderson Type I) occurred in six patients (13%); four patients had recurrent dislocations (9%; one of whom sustained a first dislocation after resection of an extensive recurrence). The first dislocation occurred after a median of 20 days (range, 1 day to 2.6 months). Patients with a single dislocation were managed with open (n = 3) or closed (n = 3) reduction. Two patients with recurrent dislocations underwent revision to a dual-mobility cup with good results; no further dislocations occurred. Others were managed with open reduction and reinforced with an attachment tube. The proportion of patients who experienced a dislocation was comparable between patients who had Type 2 (five of 21 [24%]) and Type 2–3 (five of 26 [19%]) resections (odds ratio [OR], 0.76; 95% CI, 0.19–3.09; p = 0.703). With the numbers we had we could not detect a difference in dislocation in those who had a reconstructions with (two of 16 [13%]) or without (eight of 31 [26%]) attachment tubes (OR, 0.41; 95% CI, 0.08–2.22; p = 0.301). The risk of dislocation was lower for patients with a dual-mobility cup (one of 24 [4%]) compared with those without (nine of 23 [39%]); consequently, dislocation-free survival was significantly better (HR, 0.11; 95% CI, 0.01–0.89; p = 0.038). Aseptic loosening (Henderson Type II) occurred in three reconstructions (6%). Loosening occurred in two cases with an uncemented stem (one, 57 months after fixation in a structural pelvic allograft that had failed as a result of allograft resorption; and one, 36 months after implantation with an intraoperative fracture, which had caused insufficient primary fixation) and in one with a cemented stem. Structural complications (Henderson Type III) occurred in four patients (9%); two had periprosthetic iliac fractures (one treated conservatively with a good result, one was removed as a result of infection), two had a fracture during implantation (one is discussed previously, the fracture was treated conservatively and later failed as a result of implant loosening; one was fixed with nonabsorbable sutures–the stem penetrated the iliac cortex 7 days later, for which refixation was performed; no further complications occurred). Structural failure of the implant itself was not observed.

A total of 38% (18 of 47) of our patients experienced one or more nonmechanical complications. Deep infections (Henderson Type IV) occurred in 13 patients (28%), 10 within 2 months, two after 3 months, and one after 34 months. Nine were successfully treated with surgical débridement and intravenous antibiotics. In four patients (10%; two with previous surgery–one THP, one pedestal cup), the implant was removed (three within 1 month, one after 34 months). At review, two of these patients were left flail without reconstruction and a hindquarter amputation; a Type BII rotationplasty [23] and a second LUMiC^®^ were performed in one each. Median duration of surgery was 6.5 hours (range, 4.0–13.6 hours) for patients with an infection and 5.3 hours (range, 2.8–9.9 hours) for those without (p = 0.060). Blood loss showed a statistically significant correlation with the risk of infection; blood loss was 2.3 L (range, 0.8–8.2 L) for patients with an infection and 1.5 L (range, 0.4–3.8 L) for those without (p = 0.039). Other factors we analyzed (attachment tubes, silver-coated cups) were not correlated to the risk of infection.

Local recurrence (Henderson Type V) occurred in six patients (13%; four chondrosarcomas, one clear cell chondrosarcoma, and one phosphaturic mesenchymal tumor; the latter two had tumor spill during the index procedure) after a median of 22 months (range, 10 months to 4.5 years). Five were treated with construct-sparing resections and one patient had an extensive periprosthetic recurrence; no further surgery was undertaken because of a poor prognosis. Four of 41 primary tumors metastasized (10%).

**Fig. 3:**
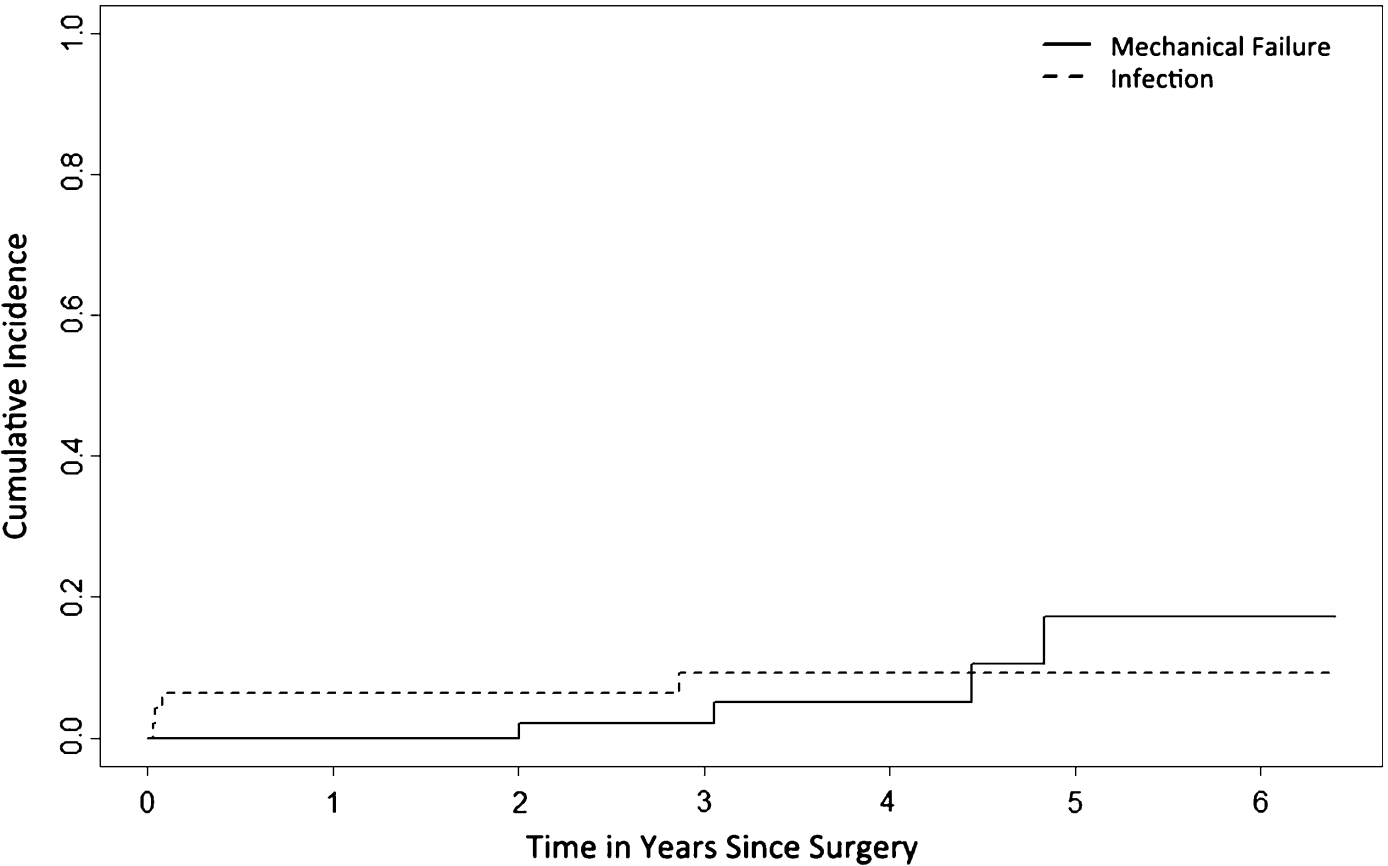
Competing risk analyses of implant failure. This plot shows the cumulative incidence of mechanical failure (Type 1–3) and infection (Type 4). Patient mortality was used as a competing event in these analyses.

The cumulative incidences of implant failure at 2 and 5 years were 2.1% (95% CI, 0–6.3) and 17.3% (95% CI, 0.7–33.9) for mechanical reasons and 6.4% (95% CI 0–13.4) and 9.2% (95% CI, 0.5–17.9) for infection, respectively (Fig. 3). Mechanical reasons for failure were instability (n = 2 [4%]; one patient underwent cup revision and was free of further complications; one patient underwent cup revision and the stem was later revised for loosening and loosening (n = 2 [4%]). Infection was the only nonmechanical failure mechanism (n = 4 [9%]). In all, 71 reoperations were performed in 25 patients (53%; range, one to eight), 59 of which (83%) were in the first postoperative year. Predominant reasons for reoperations were infection (n = 46 [65%]), mechanical reasons (n = 15 [21%]), and local recurrences (n = 6 [8%]).

Mean MSTS scores at final followup were available for 24 patients (51%). The mean score was 21 of 30 points (70%; range, 30%–93%); these were evaluated after a median of 39 months (range, 6–68 months).

## Discussion

Periacetabular resection and subsequent reconstructions pose a difficult challenge to orthopaedic oncologists. In this retrospective multicenter study, we aimed to evaluate the short-term clinical results of periacetabular reconstruction with the LUMiC^®^ prosthesis after internal hemipelvectomy for a pelvic tumor. We found that this implant is associated with a low risk of mechanical failure at short-term followup. Nevertheless, these complex reconstructions were associated with a considerable risk of complications, most notably infection.

Our study has a number of limitations. Followup duration was limited and longer term followup certainly will be needed to make any claims about intermediate- and long-term durability of this new implant. We tried to compensate for this by performing a multiinstitutional study to increase our numbers. Also, we included heterogeneous diagnoses in this study. However, patient numbers are limited and we mainly focus on the reconstruction itself rather than on oncologic outcome. In addition, as a result of the multicenter design of this study, different surgical techniques and treatment protocols have been used. A considerable number of surgeons have operated on our patients and results may have been subject to learning curves. Surgeons involved in the care of the patients were involved with data collection and reporting, which may influence the reporting of complications. We however chose to report on hard endpoints and thereby reduced the risk of assessor bias. Unfortunately, the cumulative incidence plot for implant failure does not show a clear plateau phase and further failures may be expected. We will continue to follow our patients to ascertain the role of the LUMiC^®^ in the longer term. Also, we had MSTS functional data on half of our patients, so it is possible that we have overestimated the function we might have seen if we had MSTS scores on all of the patients.

Dislocation rates were dissatisfying in the early period of our study. We were able to improve this by introducing dual-mobility articulation (one single dislocation in 24 dual-mobility cups [4%]). The results obtained with dual-mobility cups compare favorably with results previously obtained with the Pedestal cup™ prosthesis (16% recurrent dislocations, 11% single dislocation) [7] and with most other reports on periacetabular reconstruction (12%–24%) [1, 2, 15, 24, 25, 28]. Two previous authors reported comparable dislocation rates (3%–4%) [20, 33]. Our results suggest that that dual-mobility articulation may be useful for treating instability around the hip, a finding that has been reported elsewhere [27]. Currently, we use dual-mobility cups for any LUMiC^®^ reconstruction after en bloc tumor resection. Owing to the frequently massive extent of soft tissue resection, muscular function can be heavily impaired and distorted after pelvic resection. Therefore, obtaining a stable reconstruction can be difficult. In a study on 27 reconstructions with the “ice-cream cone prosthesis” (Stanmore Implants Worldwide, Elstree, UK), Fisher et al. [15] noted that dislocations occurred mainly after Type 2 or 3 resection and attributed this to the fact that virtually all muscles that attached the leg to the pelvis had been resected. The authors stated that patients should be instructed to contract their gluteal muscles before attempting to move their leg. Although we found no difference in the risk of dislocation between resection types, their “buttock-up” instruction may aid to reduce dislocation rates. We aimed to prevent dislocations by introducing an implant that would offer optimal possibilities for cup orientation and positioning and by using large-diameter femoral heads. Orientation can be difficult with the patient loosely in lateral decubitus; in experience of the leading center, computer assistance is of added value in these situations. An influence of femoral head size was not demonstrated in our study, whereas it has been reported that large-diameter heads offer advantages in terms of stability both in hip arthroplasty and pelvic reconstruction [15, 24, 30].

Loosening occurred in three reconstructions (6%): one in a patient who received uncemented fixation in a previous allograft reconstruction, one as a result of an intraoperative fracture, and one cemented stem. Our results compare favorably with the loosening rate we found in our study on the pedestal cup prosthesis (16%) [7]. On the other hand, Fisher et al. [15] reported comparable results; they described loosening in one patient with insufficient bone stock (3%). Others reported loosening of the pelvic component in 12% to 15% [1, 33]. Because the long axis of the conical stem is in line with the load-bearing axis, loading of the LUMiC^®^ causes it to anchor itself into the iliac wing. This is fundamentally different from the biomechanics of custom three-dimensional-printed or modular hemipelvic implants. Furthermore, the stem is coated with HA, which reportedly reduces the risk of loosening of uncemented implants by enhancing bony ingrowth [6]. For the aforementioned reasons, we consider this design suitable for long-term stable fixation, and we prefer uncemented press-fit fixation. Possible indications for cemented fixation include radiation, metastatic disease, and the inability to obtain rigid primary fixation.

Infection was the most common complication (28%). Although most infections (nine of 13) were successfully eradicated with débridement and antibiotics, many reoperations were performed and four reconstructions failed as a result. Previously, we reported an infection rate of 47% in reconstructions with the Pedestal cup™ prosthesis [7]. We attempted to reduce the risk of infection by introducing silver-coated cups, but with the numbers we had, we could not demonstrate an advantage with this approach. However, only the outside of the 60-mm cup was silver-coated, and limited patient numbers hampered us. It has been shown that the release of silver ions protects against infection and favorable results have been reported by others [16, 21]; future studies will need to evaluate this in greater depth. With interest we noted the promising infection rate reported by Fisher et al. [14]; three infections occurred in 27 patients (11%), and none resulted in implant failure in their short-term followup study. The authors theorized that the large amount of antibiotic-laden bone cement that they apply around the prosthesis minimizes the infection risk and allows effective treatment if it occurs. We are of the opinion that surgical duration should also be considered and, although this did not reach statistical significance, we found that the duration of surgery was greater for patients who developed an infection. This was in concordance with previous reports [18]. It is conceivable that surgical duration decreases when surgeons perform these procedures more often and in experienced teams; therefore, it might be worth considering having centralized centers that treat the majority of these patients so that patients can benefit from a team that has extensive experience in these reconstructions.

Overall cumulative incidences of implant failure at 2 and 5 years were 6.4% and 17.9%, respectively. Most studies on pelvic endoprostheses have not reported implant survival rates; however, our results compare favorably with others, reporting Kaplan-Meier estimated survival rates of 78% to 84% at 2 years [28, 33] and 40% to 60% at 5 years [25, 28].

Mean MSTS score was 70%; this is comparable with two previous studies reporting mean scores of 69% and 70% [15, 28] with either MSTS [13] or Toronto Extremity Salvage Score (TESS) [9] questionnaires. Most authors report worse functional outcome with mean scores typically ranging between 47% and 64% [2, 20, 24, 25, 33].

At short-term followup, the LUMiC^®^ prosthesis demonstrated a low frequency of mechanical complications and reoperations when used to reconstruct the acetabulum in patients who underwent major pelvic tumor resections, and we believe this is a useful reconstruction for certain periacetabular resections for tumor or failed prior reconstructions. Still, like with any type of pelvic reconstruction, complications are common after these complex procedures and we have not directly compared our patients with a similar group with a different reconstruction. Infection was the main reason for implant failure. Although the majority of the infections were eradicated with surgical débridement and antibiotics, additional ways should be sought to reduce the infection risk. Our early results are reassuring that the use of dual-mobility articulation provides for stable pelvic reconstruction in the short term. Nevertheless, future larger studies will need to confirm the durability of the construct. We will continue to follow our patients over the longer term to ascertain the role of this implant in this setting.
